# Clinical analysis and etiology of porokeratosis

**DOI:** 10.3892/etm.2014.1803

**Published:** 2014-06-24

**Authors:** CHAO-YING GU, CHENG-FENG ZHANG, LIAN-JUN CHEN, LEI-HONG XIANG, ZHI-ZHONG ZHENG

**Affiliations:** Department of Dermatology, Huashan Hospital, Fu Dan University, Shanghai 200040, P.R. China

**Keywords:** genital porokeratosis, clinical manifestation, etiology, pruritus, lesions

## Abstract

The present study was performed in order to define the clinical manifestations of porokeratosis, with particular emphasis on genital porokeratosis. A total of 55 cases of porokeratosis were retrospectively reviewed between 2000 and 2007 from Huashan Hospital (Shanghai, China). Out of 55 cases, there were 22 cases of porokeratosis of Mibelli, 17 cases of disseminated superficial actinic porokeratosis (DSAP), 15 cases of disseminated superficial porokeratosis and one case of linear porokeratosis. The ratio of males to females was 39:16. Among them, 12 cases had a family history of porokeratosis. During the five-year follow-up period, no malignant transformation was observed and no further aggravation of lesions was detected. The results indicated that the initial region of DSAP in the Chinese population may differ from Caucasians. In combination with other studies, the present study found that genital porokeratosis in the Chinese population is often associated with pruritus. Since no recurrence was observed in cases treated with surgical excision, it was suggested that surgical excision is a viable treatment strategy and should be used for porokeratotic lesions if possible. In addition, regular follow-ups are required, since the aggravation of porokeratosis may cause the development of malignancy transformation.

## Introduction

Linear porokeratosis (LP), punctate porokeratosis (PP), disseminated palmoplantar porokeratosis (DPP) and genital porokeratosis are uncommon disorders resulting from epidermal keratinization. Porokeratosis of Mibelli (PM), disseminated superficial porokeratosis (DSP) and disseminated superficial actinic porokeratosis (DSAP) are more common subtypes of porokeratosis, from which an increasing number of people suffer. Porokeratosis includes several clinical variants with a wide range of clinical presentations, which all have histologically in common the presence of a cornoid lamella. At present, several variants have been reported to be inherited as an autosomal dominant trait (1,2). According to the distribution of the lesion, porokeratosis may be classified into two types, the localized variants, including PM, LP and PP, and the extensive variants, including DSP, DSAP and DPP (1).

Although, porokeratosis was first reported more than a century ago, the etiology and pathogenesis remains unclear and the results from different studies are contradictory. Certain mutations that are associated with porokeratosis, including frameshift mutations, have been identified (2,3). However, these events are confined to certain pedigrees (4). Clinical and molecular evidence has demonstrated that porokeratosis can be considered to be a premalignant skin condition (5,6). However, effective treatments to cure porokeratosis are currently lacking. In the last twenty years, there were numerous cases of porokeratosis reported in families (2,3,7). Furthermore, there have also been a number of case reports, nevertheless, no more than 31 cases have been investigated individually.

Genital porokeratosis is extremely rare. To date, only 26 cases have been reported in previous studies (8–22). A case report of 10 cases of genital porokeratosis in Taiwan revealed that 30% had diabetes mellitus (18). More cases are required to determine whether this is associated with genital porokeratosis.

The aim of the present study was to define the clinical features of porokeratosis, with particular emphasis on genital porokeratosis, and to investigate the potential etiology. A total of 55 cases of porokeratosis were analyzed in the present study. To the best of our knowledge, this may be the largest scale survey of porokeratosis, including for genital porokeratosis.

## Subjects and methods

### Subjects

In the present study, cases were identified from Huashan Hospital (Shanghai, China). All subjects received careful examination by three dermatologists, and the diagnosis of porokeratosis was confirmed by histological examination of a skin biopsy.

### Statistical analysis

Statistical analysis was performed using SPSS version 11.0.0 (SPSS, Inc., Chicago, IL, USA). The Chi-square test was performed to analyze the effect of inheritance factors and gender on the clinical variants. The Student-Newman-Keuls test was used for the analysis of the age of onset of the clinical variant. The age of onset of the clinical variant is presented as the mean ± standard deviation. P<0.05 was considered to indicate a statistically significant difference.

## Results

### Demographics

A total of 55 cases of porokeratosis diagnosed by histopathology between 2000 and 2007 from Huashan Hospital (Shanghai, China) were reviewed retrospectively. The present study enrolled 39 males and 16 females, with an average age of 51.33 years (range, 5–84 years). Among them, 12 individuals had a family history of porokeratosis, with ≥1 first-degree relatives suffering from porokeratosis, however, the pattern of inheritance was not determined. The mean age of onset was 39.42 years (range, 5–76 years), with a mean age of onset of 25.0 years in inherited cases and 43.42 years in uninherited cases.

### Clinical manifestations

**Figure 1:**
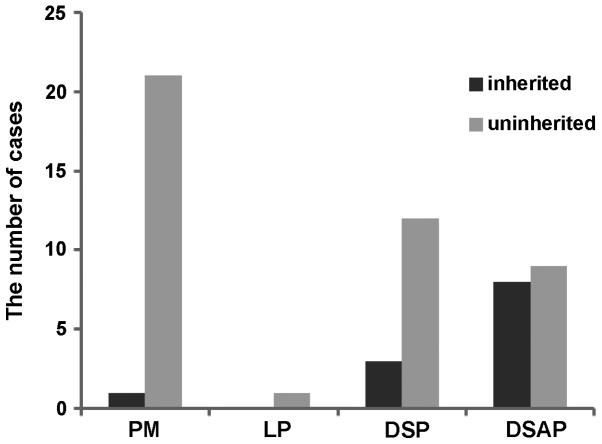
Analysis of inherited and uninherited cases of porokeratosis in the clinical variants. PM, porokeratosis of Mibelli; LP, linear porokeratosis; DSP, disseminated superficial porokeratosis; DSAP, disseminated superficial actinic porokeratosis.

**Figure 2:**
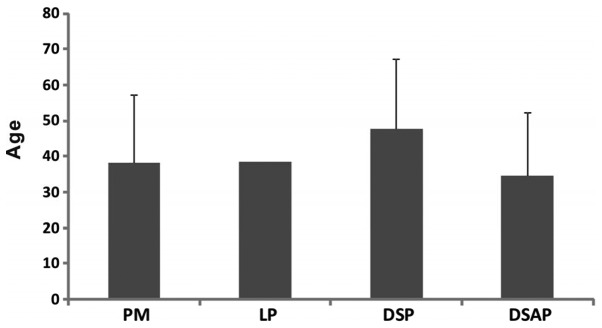
Age of onset of porokeratosis in the clinical variants. PM, porokeratosis of Mibelli; LP, linear porokeratosis; DSP, disseminated superficial porokeratosis; DSAP, disseminated superficial actinic porokeratosis.

**Figure 3:**
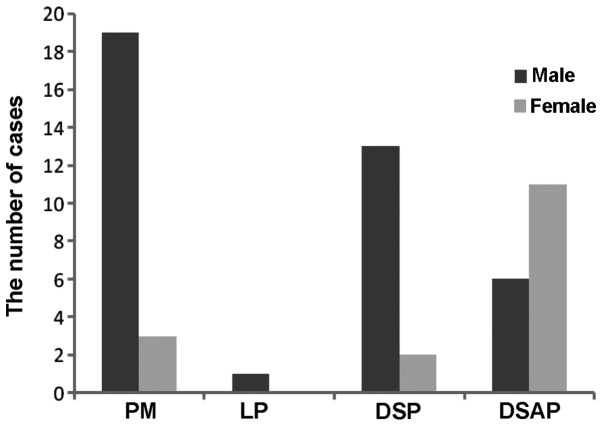
Analysis of gender in the clinical variants of porokeratosis. PM, porokeratosis of Mibelli; LP, linear porokeratosis; DSP, disseminated superficial porokeratosis; DSAP, disseminated superficial actinic porokeratosis.

In the present study, patients were classified into four clinical variants: i) 22 cases of PM; ii) 17 cases of DSAP (with the main part of the lesions developed in sun-exposed areas); iii) 15 cases of DSP; and iv) one case of LP. PP and DPP were not detected in the present study. The number of uninherited cases with PM and DSP was significantly higher than inherited cases (Fig. 1), however, no difference was observed in the DSAP cases (Fig. 1). In the analysis of the age of onset and clinical variant, no significant difference among these variants was identified (Fig. 2). The number of cases of PM and DSP was higher in males, whilst the number of cases of DSAP was higher in females (Fig. 3). The lesions initiated on the face in 17 cases, on the body in 5 cases, on the limbs in 20 cases, on the buttock in 4 cases and in the genital area in 9 cases (Table I).

In total, 40 patients investigated in the present study suffered from pruritus, with no significant difference observed between the clinical variants. Eight patients, including six patients with PM and two patients with DSP, also had verrucous hyperplasia centralized in the buttock and pubic region, and all eight patients suffered from conspicuous pruritus. The clinical features of the clinical variants are shown in Table II.

### Associated systemic conditions

Two patients with DSP had a long-term history of corticosteroid use. The lesions increased following acute renal failure in one patient with inherited DSP, with a darkening in color of the lesion observed and a raising of the hyperkeratotic margin. The lesions of one patient with PM was in burn scar tissue, whilst another patient had PM lesions in the compressed region of infected folliculitis. One patient had lesions which spread all over the body and four limbs diagnosed as DP three months after suffering from infecting viral pneumonia. One patient with genital porokeratosis also suffered from condyloma acuminatum. A total of 14 patients, including 11 patients with DSAP lesions, found that the lesions became darker and pruritus was aggravated when exposed to the sun, however, in winter, the symptoms were alleviated.

### Treatment and follow-up

Surgical excision was performed on seven patients with PM and no recurrence of porokeratosis was observed. Topical treatments were used in the remaining 48 patients, including isotretinoin, vitamin D3 and its derivatives, corticosteroid, imiquimod, hypericin and diclofenac gel.

The choice and use of the drug depended on the individual patient. During the five-year follow-up period, no noticeable aggravation of the lesions was detected.

### Genital porokeratosis

In the present study, 11 patients, 10 males and one female, suffered from genital porokeratosis (Table III). The lesions were observed in the genital area only in nine patients, whilst the other two patients suffered from lesions in the genital and adjacent areas. All 11 patients had no family history of porokeratosis. Hypertrophic plaques were found in five patients, whilst multiple papules with verrucous hyperplasia were observed in three patients. Nine patients suffered from pruritus and this symptom was exacerbated at high temperatures. One patient had a medical history of condyloma acuminatum and one patient had a medical history of folliculitis. Several cases of genital porokeratosis coexisting with genital diseases, for example condyloma acuminatum and syphilis, were reported, however, whether these diseases are associated with genital porokeratosis remains to be elucidated (18). Surgical excision was performed on two patients and no recurrence was identified during the follow-up period. The remaining nine patients received retinoic acid and corticosteroid treatment on the lesions, twice a day. The treatment relieved pruritus, however, the lesions showed no obvious disappearance. During the follow-up period, no obvious clinically suspected malignant transformation was noted and no further growth from previous lesions was observed.

## Discussion

Several family reports for porokeratosis have been previously published (2,3,23) and a considerable portion of the reports described DSAP families. Our previous studies demonstrated that mutations in SSH1 or ARPC3 are responsible for DSAP in certain pedigrees (2,3). Furthermore, in the present study, it was observed that among the inherited cases (12 cases), eight cases were DSAP, which accounted for 66.7% of the inherited cases. Therefore, this suggests that inheritance may potentially be important in DSAP, however, the potential genetic mechanisms of DSAP remain to be elucidated. As with other inherited diseases, inherited cases of porokeratosis had a significantly earlier average age of onset (25.08 years), compared with sporadic cases (43.42 years).

Previous studies have demonstrated that males more frequently suffer from porokeratosis, compared with females (1,24). A total of 39 males and 16 females were enrolled in the present study. It is generally accepted that PM is more common in males than in females (1), and the results from the present study suggest that this may also be true for DSP.

Porokeratosis may involve every part of the body, including the oral mucosa. According to previous studies, PM is usually confined to the four limbs (25). However, in the cases included the present study, the initial region was primarily the buttock and genital area. Previous studies have also found that the initial region of DSAP was usually the skin often exposed to sunlight, but rarely the face. By contrast, in the present study, it was observed that 82.4% of cases with DSAP lesions originated from the face. A family report of DSAP in China with 100 cases described that the lesions all originated from the face (23). This suggests that the initial region of origin of DSAP in the Chinese population is the face, which differs from the results observed in Caucasians (26).

In eight cases, including six PM and two DSP, verrucous hyperplasia of plaques and multiple papules were observed associated with obvious pruritus (Table II). This suggested that verucous hyperplasia may be caused by friction, scratching, long-term compression, partial moisture and chronic diseases.

The etiology and pathogenesis of porokeratosis are complex and multifactorial (27). At present, genetic factors, ultraviolet light, trauma, immunosuppression and infectious agents have been implicated in porokeratosis (27). A prospective study revealed that 10.68% of renal transplant recipients (103 patients in total) suffered from porokeratosis (28). In the present retrospective analysis, two patients developed lesions following long-term systemic corticosteroids treatment for allergic asthma and chronic nephritis. In addition, in one patient with inherited porokeratosis, the lesions aggravated following acute renal failure. These cases imply that immune disorders may be associated with porokeratosis.

Although no direct association between porokeratosis and ultraviolet light has been verified, it is generally accepted that exposure of lesions to ultraviolet light should be avoided. In the present study, in 14 cases, including 11 cases of DSAP, it was reported that pruritus was aggravated, along with a darkening of the lesions, when lesions were exposed to sunlight. In the winter, the symptoms were alleviated. A previous study has suggested that ultraviolet light may affect the immune function in localized skin (29). In any case, it is clear that exposure of lesions to ultraviolet light should be avoided (29–31).

Certain studies have revealed that porokeratosis may also be caused by burns and infection (32,33). In the present study, it was observed that porokeratosis occurred in one patient with burn scar tissue, one patient suffering from folliculitis and another patient suffering from viral pneumonia. Surgical removal of the lesions was performed in these cases and no recurrence was observed.

In the present study, all the patients suffering from genital porokeratosis had no family history of porokeratosis and no obvious contributing factor was identified. An analysis of all the reported cases of genital porokeratosis (29 previously reported cases and 11 cases reported in the present study) found that 65% of patients were Chinese and 92.5% of patients were males (18–21,34,35). However, whether the Chinese population and males are more susceptible to genital porokeratosis requires further investigation. A retrospective analysis of 10 cases performed in Taiwan suggested that the humid climate may be associated with genital porokeratosis (18). It was found that 78.4% of cases had pruritus and 62.2% of cases had hyperplastic lesions. Compared with cases from previous studies, the present study found that the majority of cases in China suffered from pruritus, however, the majority of cases in western countries did not (8,20).

Due to its distinct clinical characteristics, it has been suggested that genital porokeratosis should be classified as a new clinical variant (18). According to the characteristics and the region of the lesions, five subtypes of variations in morphology in the 11 cases of genital porokeratosis were observed: : i) classical porokeratosis of Mibelli, with classical round-shaped lesions, characterized by atrophic patches surrounded by an elevated border, which was often distributed in the penis, scrotum and pubic region; ii) hyperkeratotic variant, characterized by a thickening of the central region and a slightly raised hyperkeratotic border, often distributed in the perianal region and buttock; iii) porokeratosis ptychotropica (36), characterized by butterfly-like plaques, with verrucous hyperplasia, primarily distributed on the buttock, however, occasionally located in other wrinkling regions; iv) ulceroproliferative porokeratosis, with ulcerative round-shaped plaques, often distributed on the penis and scrotum; v) verrucous hyperplasia, characterized by keratotic and hypertrophic plaques and nodules in the genital region.

Due to similar clinical manifestations, genital porokeratosis may easily be misdiagnosed as psoriasis, verrucous tuberculosis of the skin, verrucous lichen planus, syphilis and condyloma acuminatum.

The rate of malignant transformation is high in PM, (~6.8–11% of cases) (1). If the lesions spread excessively, surgery, laser treatment and cryotherapy are suggested. Surgery is the most effective treatment; however, due to the size, number and location of the lesions surgery may not always be possible. In the present study, surgical excisions were performed in seven cases and, during the five-year follow-up period, no recurrence was observed. For cases where surgery is unavailable, the use of tropical drugs and other treatment modalities, including laser treatment and cryotherapy, are required as well as regular follow-up care in order to manage and control the disease. In cases where malignant transformation was detected, localized surgery was able to be performed.

In conclusion, it was observed that DSAP may originate from the face in the Chinese population, which differs from what is observed in Caucasians. The present study also found that genital porokeratosis was often associated with pruritus. Since no recurrence of porokeratosis was observed following surgical excision, this suggests that surgical excisions may completely cure porokeratosis. Therefore, it is suggested that surgical excisions should be performed for the treatment of porokeratotic lesions if possible and regular follow-up is required, given that malignancy is possible in porokeratosis.

## Figures and Tables

**Table I:** Analysis of the initial regions of the lesions from different clinical variants of porokeratosis.

				Initial region			
				
Clinical type	Number of cases	Age of onset, range (years)	Face, n (%)	Body, n (%)	Limb, n (%)	Buttock, n (%)	Genital area, n (%)
PM	22	22–76	2 (9.09)	1 (4.55)	7 (31.82)	1 (4.55)	11 (50.00)
LP	1	39	0 (0.00)	1 (100)	0 (0.00)	0 (0.00)	0 (0.00)
DSP	15	16–68	1 (6.67)	3 (20)	10 (66.67)	1 (6.67)	0 (0.00)
DSAP	17	5–52	14 (82.35)	1 (5.88)	2 (11.76)	0 (0.00)	0 (0.00)

PM, porokeratosis of Mibelli; LP, linear porokeratosis; DSP, disseminated superficial porokeratosis; DSAP, disseminated superficial actinic porokeratosis.

**Table II:** Clinical manifestations of the clinical variants.

Clinical variant	Number of cases	Obvious keratotic border, n (%)	Verrucous hyperplasia n (%)	Pruritus n (%)
PM	22	15 (68.18)	6 (27.27)	19 (86.36)
LP	1	0 (0.00)	0 (0.00)	1 (100)
DSP	15	12 (80)	2 (13.33)	11 (73.33)
DSAP	17	11 (64.71)	0 (0.00)	9 (52.94)

PM, porokeratosis of Mibelli; LP, linear porokeratosis; DSP, disseminated superficial porokeratosis; DSAP, disseminated superficial actinic porokeratosis

**Table III:** Patient demographics for genital porokeratosis.

Patient	Gender	Age of onset (years)	Course of disease (years)	Affected regions	Symptoms	Medical History	Clinical manifestation
1	Male	8	1	Perianal region	Pruritus	No	Reddish plaque (1.5×10 cm) with raised border
2	Male	22	8	Scrotum and penis	Pruritus	No	Verrucous papule
3	Female	25	6	Labia majora	Pruritus	No	Verrucous papule (3×4 mm)
4	Male	28	5	Scrotum and penis	Pruritus	No	Multiple verrucous papules 3×3 mm
5	Male	26	8	Perianal region	Pruritus	No	Reddish plaques with raised borders
6	Male	36	10	Scrotum	Pruritus	Condyloma acuminatum	Reddish plaque (1.5 cm) with raised border
7	Male	52	3	Perianal region	No	No	Reddish papule (3×4 mm)
8	Male	48	8	Perianal region	Pruritus	No	Reddish and keratotic plaque (1×3 cm)
9	Male	61	2	Perianal region and buttock	Pruritus	Folliculitis	Multiple reddish papules with raised borders
10	Male	50	18	Perianal region and buttock	Pruritus	No	Multiple reddish plaques with raised borders
11	Male	50	34	Scrotum	No	No	Keratotic plaque (4 mm)
